# Multifunctional Polymer Nanocomposites

**DOI:** 10.3390/ma19061165

**Published:** 2026-03-17

**Authors:** Yinan Zeng, Jianhua Zhang

**Affiliations:** 1 Department of Polymer Science and Engineering, School of Chemical Engineering and Technology, Tianjin University, Tianjin 300350, China; zyn757810425@tju.edu.cn; 2 Tianjin Key Laboratory of Membrane Science and Desalination Technology, Tianjin University, Tianjin 300350, China

Polymer nanocomposite materials are a type of composite material that physically or covalently incorporates nanosized particles or nanostructures into a matrix of cross-linked polymer networks. The polymer nanocomposites integrate the inherent flexibility, processability, and chemical stability of polymer matrices with the unique physicochemical, mechanical, and functional properties of nanoscale fillers (e.g., carbon nanotubes, graphene, metal oxides, layered silicates, and quantum dots) [1,2,3,4,5,6,7,8,9]. The incorporation of nanomaterials into polymer matrices can render new functionalities to composites and produce superior physicochemical properties absent in individual components, offering an efficient route to enhance the physicochemical properties and thus expand the application scopes of both polymer materials and nanomaterials [10,11,12,13]. Owing to their versatile and customizable characteristics, multifunctional polymer nanocomposites have found widespread and rapidly expanding applications across numerous high-tech fields, including aerospace, electronics and optoelectronics, automotive engineering, biomedicine, environmental engineering, and smart textiles [14,15,16,17,18,19,20,21,22]. This Special Issue gathers and highlights recent research achievements in the design, fabrication, characterizations and applications of multifunctional polymer nanocomposites. This Special Issue includes eight papers in total, of which five are research articles and three are review papers, which are expected to be able to serve as a bridge for academic exchange and technological innovation in the field of polymer nanocomposites. The contributions of this collection are summarized below.

The key characteristics of multifunctional polymer nanocomposites stem from the synergistic coupling of polymer matrices and nanofillers, manifesting in a remarkable combination of enhanced mechanical, thermal, electrical, optical, magnetic, and barrier properties—even at ultra-low nanofiller loadings (typically <5 wt%). Thermally, these nanocomposites exhibit elevated glass transition temperatures, thermal decomposition temperatures, and thermal conductivity, addressing the intrinsic thermal instability of many pristine polymers. The enhanced thermostability of polymer nanocomposites can ensure the efficient exploration of the oil and gas resources under harsh conditions featuring high temperature, high pressure, and high salinity in deep formations. The paper of Zhang et al. [23] comprehensively summarized the design, preparation and applications of polymer nanocomposites as additives in drilling operation. Particular focus was placed on the strategies and structure–property–performance relationships of polymer nanocomposites to enhance the performance of drilling fluid under high-temperature, high-pressure, and high-salinity conditions in the deep formation. Future perspectives for achieving excellent comprehensive performance are also discussed to motivate future contributions and explore new possibilities. Polypyrrole (PPy) as a kind of biocompatible conductive polymer has excellent conductivity, excellent redox properties, biocompatibility, easy synthesis, and environmental stability, which has potential applications in fields such as biomedical systems, flexible electronics, and bioelectronics. Zhao et al. [24] summarized the research progress of electrospun PPy nanocomposite fiber materials and their related applications in the field of tissue engineering. They comprehensively analyzed the topological characteristics of three composite fiber architectures—randomly distributed, aligned, and core–shell structures—and elucidated their application mechanisms in nerve regeneration, skin repair, bone mineralization, and myocardial tissue reconstruction (e.g., facilitating oriented cell migration and regulating differentiation through specific signaling pathway activation).

The three-dimensional hydrogels and nanomaterials have shown a very high potential for medical therapeutic and diagnostic applications. However, some inherent limitations of hydrogels and nanomaterials inhibited their widespread applications. The incorporation of nanomaterials in a three-dimensional polymeric hydrogel matrix as an innovative means to obtain nanocomposite hydrogels with improved properties and multiple functionalities has gained significant attention in the biomedical field. The review of Chen et al. [25] presented a comprehensive overview of the integration of functionalized hydrogels and flexible electronics from the viewpoint of materials science, investigating their promising applications as well as the technical challenges involved in treating diabetic wounds. Additionally, it proposes potential future research pathways, aiming to establish a theoretical basis for facilitating technological advancement and clinical application in this domain. Li et al. [26] fabricated a hydrogel by grafting 4-formylphenylboronic acid (FPBA) onto carboxymethyl chitosan (CMCS) and mixed it with oxidized sodium alginate (OAlg) to form a dual-dynamic covalent hydrogel (CPOA), which can effectively load exosomes and form hydrogel nanocomposites (CPOA@Exos). The CPOA@Exos can not only show good injectability and self-healing ability due to its dual-dynamic covalent crosslinking network, but also sustainedly release exosomes at the diabetic wound site while effectively scavenging excess reactive oxygen species (ROS). The CPOA@Exos system as a promising therapeutic strategy for the management of diabetic wounds was demonstrated to be able to modulate inflammatory responses in diabetic wounds, promote macrophage polarization toward the M2 phenotype, and accelerate wound repair. In Kalska-Szostko et al. [27], they first prepared copper and silver nanoparticles through an eco-friendly green chemistry method using beetroot extract. And then they demonstrated that the green-synthesized copper and silver nanoparticles can be effectively embedded into a polypropylene polymer matrix, while maintaining their physicochemical stability and antibacterial performance. Importantly, the nanocomposites incorporating green-synthesized copper nanoparticles displayed remarkably improved bactericidal and fungicidal effects relative to those containing silver nanoparticles or copper nanoparticles fabricated via traditional chemical reduction techniques. These findings verify that the synthetic route exerts a critical influence on determining the biological activity of the final polymer nanocomposites.

The multifunctional polymer nanocomposites have also been developed for drug and gene delivery systems, tissue engineering scaffolds, biosensors, and medical imaging contrast agents. The paper of Wang et al. [28] synthesized a series of core–shell-structured calcium peroxide/poly(ethylene glycol)@silica (CPO@SiO_2_) nanoparticles with tunable oxygen-generating properties. The nano-CPO core acts as the oxygen source to improve hypoxia, while the poly(ethylene glycol) and SiO_2_ shell layer serves as the physical barrier to improve biocompatibility and control the oxygen-generating rate. As a result, the CPO@ SiO_2_ hybrid nanoparticles can support cell survival under hypoxia, effectively decrease oxidative stress damage, and reduce the levels of expression of hypoxia-induced superoxide dismutase and malondialdehyde for repairing damaged tissue. Yang et al. [29] synthesized N-doped graphene quantum dots (N-GQDs) via a one-step hydrothermal route using cellulose as the starting material and ethylenediamine as the nitrogen dopant. The entire procedure eliminated the use of strong acids or bases and prevented contamination from inorganic salt byproducts and heavy metal ions. The as-synthesized N-GQDs exhibited excellent photostability and salt tolerance. And then they prepared a new fluorescent sensing platform with high selectivity and sensitivity for the detection of 6-mercaptopurine (6-MP) via the fluorescence inner filter effect of N-GQDs. Furthermore, this probe can be effectively applied to the determination of 6-MP in human urine samples, verifying that the N-GQD-based fluorescent sensor possesses outstanding practical application potential.

The introduction of inorganic nanomaterials as fillers into polymer matrixes can endow the resulting polymer nanocomposites with unique mechanical properties, optical properties, magnetic properties, and/or electrical properties. The paper of Iritani et al. [30] developed highly functional zinc oxide nanoparticles (ZnONPs) by surface modification with 6-amino-1-hexanol bearing both amino and hydroxyl functional groups. The composite films of polylactic acid (PLA) reinforced with the functionalized ZnONPs were prepared and then evaluated. The composite containing functionalized ZnONPs showed greater tensile strength in comparison to films loaded with unmodified ZnONPs. Moreover, the elevated water contact angle values suggested that surface modification improved the hydrophobic characteristics of the composite films. This work validated the feasibility of using surface-modified ZnONPs as reinforcing additives, underscoring an effective strategy for the design of advanced eco-friendly materials.

In summary, multifunctional polymer nanocomposites have emerged as a cornerstone of advanced material science, driven by their unique fabrication flexibility, synergistic multi-property characteristics, and broad application potential. With the continuous advancement of nanotechnology, filler modification strategies, and composite processing techniques, the design and synthesis of polymer nanocomposites with more precise and diverse functionalities will continue to evolve. Future research directions will focus on developing sustainable and bio-based polymer matrices and nanofillers, scaling up fabrication processes with high efficiency and low cost, and exploring novel multifunctional combinations to address emerging challenges in energy, environment, healthcare, and advanced manufacturing. As such, these materials will play an increasingly pivotal role in driving technological innovation and sustainable development across global industries.

## Data Availability

Not applicable.
